# Characterizing athletic healthcare: A perspective on methodological challenges, lessons learned, and paths forward

**DOI:** 10.3389/fspor.2022.976513

**Published:** 2022-08-29

**Authors:** Kenneth C. Lam, Alison R. Snyder Valier, Tamara C. Valovich McLeod, Ashley N. Marshall

**Affiliations:** ^1^Department of Interdisciplinary Health Sciences, Arizona School of Health Sciences, A.T. Still University, Mesa, AZ, United States; ^2^Department of Athletic Training, Arizona School of Health Sciences, A.T. Still University, Mesa, AZ, United States; ^3^School of Osteopathic Medicine in Arizona, A.T. Still University, Mesa, AZ, United States; ^4^Department of Rehabilitation Sciences, Beaver College of Health Sciences, Appalachian State University, Boone, NC, United States

**Keywords:** electronic medical record (EMR), electronic health record (EHR), athletic training, observational research, clinical data analysis

## Abstract

Recently, there has been an emphasis on collecting large datasets in the field of sports medicine. While there have been great advances in areas of sport performance and sport epidemiology, there have been fewer efforts dedicated to understanding the effectiveness and impact of athletic healthcare, including injury prevention programs and rehabilitation interventions provided at the point-of-care. In 2009, the Athletic Training Practice-Based Research Network (AT-PBRN) was launched to address this need, with the mission of improving the quality of care provided by athletic trainers. Unlike other research efforts in sports and medicine, such as sport epidemiology, there are fewer methodological best practices specifically related to clinical data in athletic healthcare. As a result, the AT-PBRN has encountered several methodological challenges during its tenure and has established guidelines based on various sources within the fields of sports and medicine to address these challenges. Therefore, the purpose of this perspective is to identify the challenges and describe strategies to address these challenges related to characterizing athletic healthcare using a large database. Specifically, challenges related to data entry (data quality and reliability) and data extraction and processing (data variability and missing data) will be discussed. Sharing challenges and perspectives on solutions for collecting and reporting on athletic healthcare data may facilitate a greater consistency in the approach used to collect, analyze, and report on clinical data in athletic healthcare, with the goal of improving patient outcomes and the quality of care provided by athletic trainers.

## Introduction

Recently, there has been an emphasis in athletic healthcare on collecting large datasets to generate evidence, support clinical decisions, and enhance patient outcomes (Sauers et al., 2012; Dompier et al., 2015). Although large datasets have supported efforts in sport performance (Seshadri et al., 2019a,b) and sport epidemiology (Kerr et al., 2018; Bohr et al., 2021), there have been fewer efforts aimed at understanding the overall impact of athletic health care, including the effectiveness of injury prevention programs and rehabilitation techniques provided by healthcare providers. Point-of-care data collected *via* electronic records provide valuable insights into these important lines of inquiry by characterizing the care provided to patients and generating point-of-care evidence (Casey et al., 2016; Cowee and Simon, 2019; Lam et al., 2020; Marshall and Lam, 2020).

Due to the limited efforts related to the use of electronic records data in the athletic healthcare, there are relatively few methodological best practices established specifically for the field (Kerr et al., 2018; Bohr et al., 2021). Furthermore, as investigations using clinical data evolve and become more intricate, new methodological challenges arise that need to be appropriately addressed. Luckily, while these challenges are new in athletic healthcare, other healthcare disciplines have encountered similar problems and can offer appropriate strategies by which to address these issues (Benchimol et al., 2015; Casey et al., 2016; Cowie et al., 2017). The purpose of this perspective is to identify the challenges related to the use of electronic records data in athletic healthcare, offer strategies by which to address these challenges, and discuss paths forward in this relatively new area of research in athletic healthcare. Our perspectives are based on our experiences managing the Athletic Training Practice-Based Research Network (AT-PBRN), a network of researchers and clinicians connected *via* a web-based electronic medical record (EMR), and comments we have received from other scientists during the peer-review process of our publications.

## Electronic records data: The clinician as a data collector

The use of electronic records such as electronic medical records (EMRs) and electronic health records (EHRs) has been well-documented in the global healthcare system (Casey et al., 2016; Cowie et al., 2017; Rudin et al., 2020). Efforts to design common electronic health record systems that cross healthcare organizations and practices have been promoted in part because these point-of-care data can answer important clinical questions and advance the science related to patient care (Casey et al., 2016; Cowie et al., 2017). Central to leveraging clinical data for research purposes is positioning the clinician as a primary contributor to the scientific process. Not only can clinicians collect point-of-care data *via* electronic records but they can provide valuable and important insights into current practices and challenges occurring at the point-of-care that can help guide targeted research efforts (Sauers et al., 2012).

The healthcare professional that is optimally situated to fulfill this position within the realm of athletic healthcare is the athletic trainer (AT). Similar to athletic therapists and physiotherapists, ATs specialize in physical medicine and rehabilitation sciences and are frequently the first healthcare professional to manage injuries or illnesses arising from physical activity including sprains, fractures, concussions and life threatening conditions such as exertional heat illness, spinal cord injury, and cardiac arrest (National Athletic Trainers' Association). In the United States, ATs practice under the direction of a physician and typically provide on-site care in various patient care settings such as intermediate schools, secondary schools, colleges, clinics, professional sports, military, and industrial facilities (National Athletic Trainers' Association). Due to their unique on-site availability, ATs are easily accessible to their patients and can manage their patients throughout the duration of medical care, from the pre-injury stage through rehabilitation to the return to participation. In fact, most ATs have the latitude to treat their patients on a daily basis from time of injury to discharge. Due to these unique aspects of athletic training clinical practice, ATs are in an excellent position to collect clinical data that would elucidate various aspects of athletic healthcare including injury prevention and rehabilitation.

To gather point-of-care data from ATs, researchers from A.T. Still University launched the AT-PBRN in 2009 (Valovich McLeod et al., 2012), the first and only Agency for Healthcare Research and Quality (AHRQ) recognized practice-based research network in athletic training. In 1999, the AHRQ was charged by the United States Congress with the support and oversight of these networks to aid in the evolution of point-of-care clinical research. As part of that oversight, the AHRQ established specific infrastructure criteria for PBRNs to be recognized and to receive federal grant funding (Green et al., 2005). The mission of the AT-PBRN is to improve the patient outcomes and enhance the quality of care for patients under the care of ATs (Valovich McLeod et al., 2012). To achieve its mission, the AT-PBRN developed a Health Insurance Portability and Accountability Act (HIPAA) compliant electronic medical record (CORE-AT EMR) that connects clinicians and researchers (Valovich McLeod et al., 2012). Currently, the AT-PBRN consists of over 90 ATs providing care in a variety of settings (e.g., secondary schools, colleges, professional sports, industrial, military) across 35 states in the United States.

**Figure 1:**
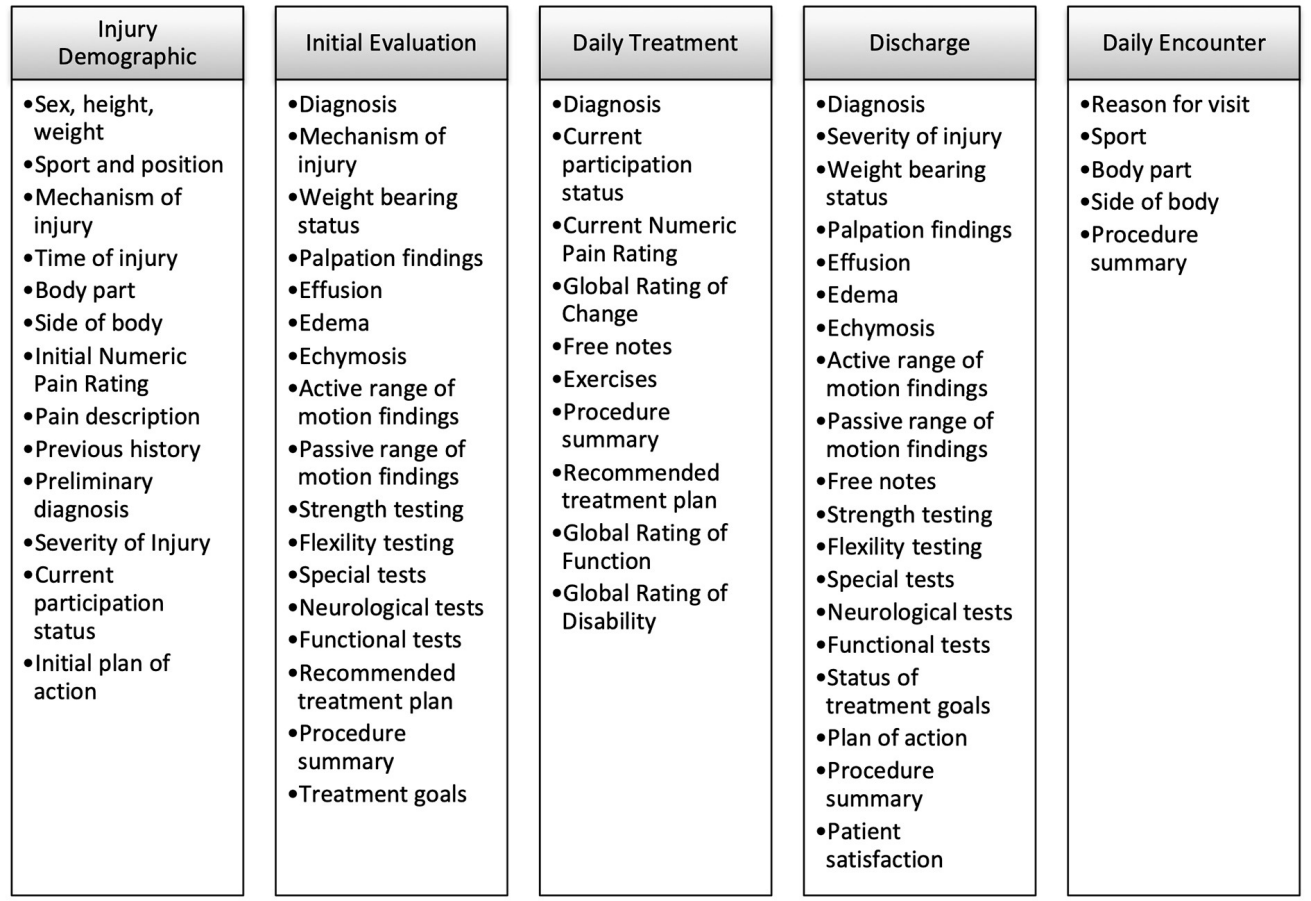
Primary forms within the electronic medical record with associated variables.

As a clinical tool, the EMR provides an efficient way for ATs to document their routine patient care through a variety of forms (Figure 1) and patient-reported outcome measures. As with any EMR, the data entered into the system can be viewed and accessed by the AT who recorded those data for their patients. However, the data are also de-identified and aggregated into a large, centralized database for research purposes (Valovich McLeod et al., 2012). Since its inception, the AT-PBRN has demonstrated the ability to reliably collect clinical data at a rapid pace, generate large and diverse datasets, and produce data that are generalizable to routine patient care (Valovich McLeod et al., 2012, 2019; Lam et al., 2015; Lam et al., 2016b, 2021, 2022; Marshall et al., 2019). To date, the AT-PBRN has recorded over 40,000 injuries, 325,000 treatments, and 11,000 patient-reported outcome measures.

Using this large dataset, the AT-PBRN research team has been able to describe various aspects of athletic training clinical practice. Specifically, early efforts from the AT-PBRN have described patient (e.g., sport, diagnosis), treatment (e.g., type, amount, and duration of treatment), and value (e.g., cost, quality) characteristics of athletic training clinical practice (Valovich McLeod et al., 2012, 2019; Lam et al., 2015; Lam et al., 2016b, 2021, 2022; Marshall et al., 2019). Specifically, these investigations have provided evidence to support that over 60% of daily patient encounters are preventative in nature (Lam et al., 2016a), over 85% of patients report a meaningful change in patient outcomes within 2 weeks of an ankle sprain injury (Lam et al., 2022), and the average estimated cost of care for patients following ankle (Marshall et al., 2019) and knee (Lam et al., 2021) injuries. Although these results can be viewed as modest, these findings have historically eluded athletic healthcare professionals and are essential for identifying effective injury prevention and treatment strategies. For instance, clinicians can evaluate whether the preventative services they are providing are reducing the incidence of injury, observe trends in specific outcomes, and identify the costs associated with athletic training services in concert with patient outcomes to support meaningful and cost-effective treatment.

While the AT-PBRN has collected relevant data and disseminated meaningful information, it has also encountered methodological challenges during its existence. Thus, the AT-PBRN has refined its approach to data collection and reporting over its tenure, frequently relying on methodological practices from related fields (Benchimol et al., 2015; Kerr et al., 2018). Since much of our refinement process has been guided by other scientists during the peer-review process of our previously published manuscripts (Valovich McLeod et al., 2012, 2019; Lam et al., 2015; Lam et al., 2016b, 2021, 2022; Marshall et al., 2019), we felt it important to share our experiences with the athletic healthcare community in effort to further support scientific progress within the area of electronic records data use in athletic healthcare.

## Use of EMR data: Lessons learned and paths forward

Although EMRs can support prospective (e.g., randomized controlled trial) and retrospective (e.g., observational designs) investigations (Cowie et al., 2017; Marshall and Lam, 2020), we will primarily focus our discussion on the observational design since most of our efforts to date have used this approach (Valovich McLeod et al., 2012, 2019; Lam et al., 2015, Lam et al., 2016b, 2021, 2022; Marshall et al., 2019). In brief, observational data are usually recorded during routine, real-life encounters (Concato et al., 2010). Importantly, in contrast to experimental designs, variables from observational investigations are collected prospectively, rarely manipulated or controlled, and analyzed retrospectively (Concato et al., 2010). For example, findings from a recent observational study on the clinical presentation of patients with ankle injuries (Marshall et al., 2019), data were reported as they were recorded by the AT. Due to these characteristics, observational studies are thought to offer data that are more representative of the target population and more generalizable to the larger patient population as compared to experimental designs (Concato et al., 2010). In contrast, an experimental study modifies routine clinical practice in some way by incorporating an intervention to be evaluated. More formally, while experimental designs generally offer higher levels of internal validity but lower external validity, observational designs typically offer greater external validity but lower internal validity (Lam et al., 2020). Because of this, safeguards should be in place to optimize data collection and entry in observational designs.

### Data entry: Data quality and reliability

#### Challenges

A challenge in many observational designs is ensuring data quality and reliability. To optimize consistency across multiple clinicians and multiple sites, it is vital to have a comprehensive training program in place for all clinicians. The training program should include components such as the overall purpose and objectives of the data collection effort, the proper use of the EMR, and operational definition of targeted variables. The goal of these measures is to ensure that data are collected in a reliable and consistent manner and optimizes data quality. Within the AT-PBRN, we require clinicians to complete an on-boarding process that includes a 2-h training program addressing all of the major components listed above, prior to use of the EMR (Valovich McLeod et al., 2012). Although we had the necessary training procedures in place, we encountered other challenges that impacted data entry.

An early goal of the AT-PBRN was to collect as many clinical variables as possible. Our rationale was that more variables would produce the greatest ability to answer various clinical questions and increase the utility of the AT-PBRN. Thus, most variables within the EMR required an entry before an EMR form (Figure 1) could be submitted into the system. The volume of variables produced practical challenges. For example, if an AT was completing an injury evaluation form but forgot to ask the patient about a required field (e.g., known allergies) the AT could not submit the form to the EMR. In addition, in its initial version, the EMR focused primarily on time-loss injuries or injuries that required a comprehensive injury evaluation. However, if a patient visited the clinic for prevention purposes (e.g., an injury prevention program, maintenance program), there was not a dedicated form by which to capture those encounters. These two situations resulted in decreased usability of the EMR for the clinician and missed opportunities to collect relevant clinical data.

#### Lessons learned and paths forward

Over time, we recognized the focus on gathering as many variables as possible it can be at the expense of securing consistent users of the EMR. Our group also understood the importance of soliciting feedback from clinicians to improve the EMR user interface. Our on-going philosophy is that the more user friendly the EMR is, the more comprehensive and consistent data collection will be which speaks to the quality and reliability of the data. While these measures are well-known and are part of best practices related to observational studies, current literature focuses less on the importance of soliciting clinician feedback throughout the data collection process.

By soliciting clinician feedback, we have made important changes to the existing fields and content of forms in the EMR, which have facilitated better usability and data entry. For instance, fewer fields are now required within the EMR. The AT-PBRN research team reviewed all forms within the EMR to identify essential vs. non-essential variables and used feedback from clinicians to support these decisions. This is an on-going process as the identification of required fields within the EMR is guided by both clinician feedback and on-going research efforts. For example, a current prospective research study on the effectiveness of ankle treatments requires clinicians to enter more variables related to the specific aspects of care (e.g., must identify specific manual therapy techniques used) than are required when documenting care for other injuries.

One of the most significant changes within the EMR based on clinician feedback was the development of a daily encounters form (Figure 1), which allowed for the entry of non-time loss or non-injury patient encounters (Lam et al., 2016b). Interestingly, the addition of the daily encounters form resulted in the collection and publication of the largest dataset to date from the AT-PBRN and offered findings to better capture the important role ATs play in providing preventative services to their patients (Lam et al., 2016b). Giving clinicians the opportunity to document these patient encounters provide a more complete perspective on athletic training clinical practice. Importantly, these efforts highlight the important role clinicians can play in guiding research efforts and how point-of-care insights from clinicians can help generate valuable point-of-care evidence.

### Data extraction and processing: Data variability and missing data

#### Challenges

As with many observational designs, data variability and missing data are common challenges. These challenges are further compounded within the context of athletic training practice. Unlike other, more traditional healthcare delivery models, the practice of athletic training can be highly variable. For example, in traditional delivery models of physical medicine, patients may follow structured appointment schedules for their condition as approved by their health insurer (e.g., a number of visits spread over weeks). In contrast, since ATs typically work on-site with their patients and do not function under an insurer model, patients may seek treatment as needed which often translate to multiple visits a week for prevention, treatment, or maintenance services. This could mean that one patient seeks and receives treatment every day while another patient seeks and receives an initial evaluation and then does not return for additional care. Further, there could be any number of different combinations of treatment frequency and duration for similar patient cases. The variability in delivery of care makes it difficult to aggregate data at similar time points across like-patients. For example, understanding patient outcomes related to the care over time is desired to demonstrate the impact that AT services have on patients from injury to return to sport. However, consistency in time points for outcomes administration is so varied that aggregating in a meaningful manner is a challenge.

#### Lessons learned and paths forward

Missing data and data variability were problematic for various retrospective studies characterizing athletic training practice. For example, we recently aimed to estimate costs associated with the management of ankle (Marshall et al., 2019) and knee (Lam et al., 2021) injuries. When extracting data, we found a fair number of patient cases that were entered into the EMR but not formally discharged from care. These missing time points were likely due to patients who were lost to follow-up, a trend we found in a previous descriptive study (Valovich McLeod et al., 2019). However, in economic analyses, it is important to provide as close an estimate of costs as possible. As a result, we considered what a typical complete patient case should consist of and used those criteria to identify inclusion criteria for the study. A complete patient case was defined as one that had documented (1) first encounter or injury demographics form, (2) injury evaluation form, (3) daily treatment forms with at least one encounter per week for the duration of care, and (4) a discharge form (Figure 1) (Marshall et al., 2019; Lam et al., 2021). By establishing criteria to fairly represent a typical complete patient case, findings from these investigations likely provide closer estimates of direct costs associated with the management of ankle and knee injuries by ATs (Marshall et al., 2019; Lam et al., 2021).

**Figure 2:**
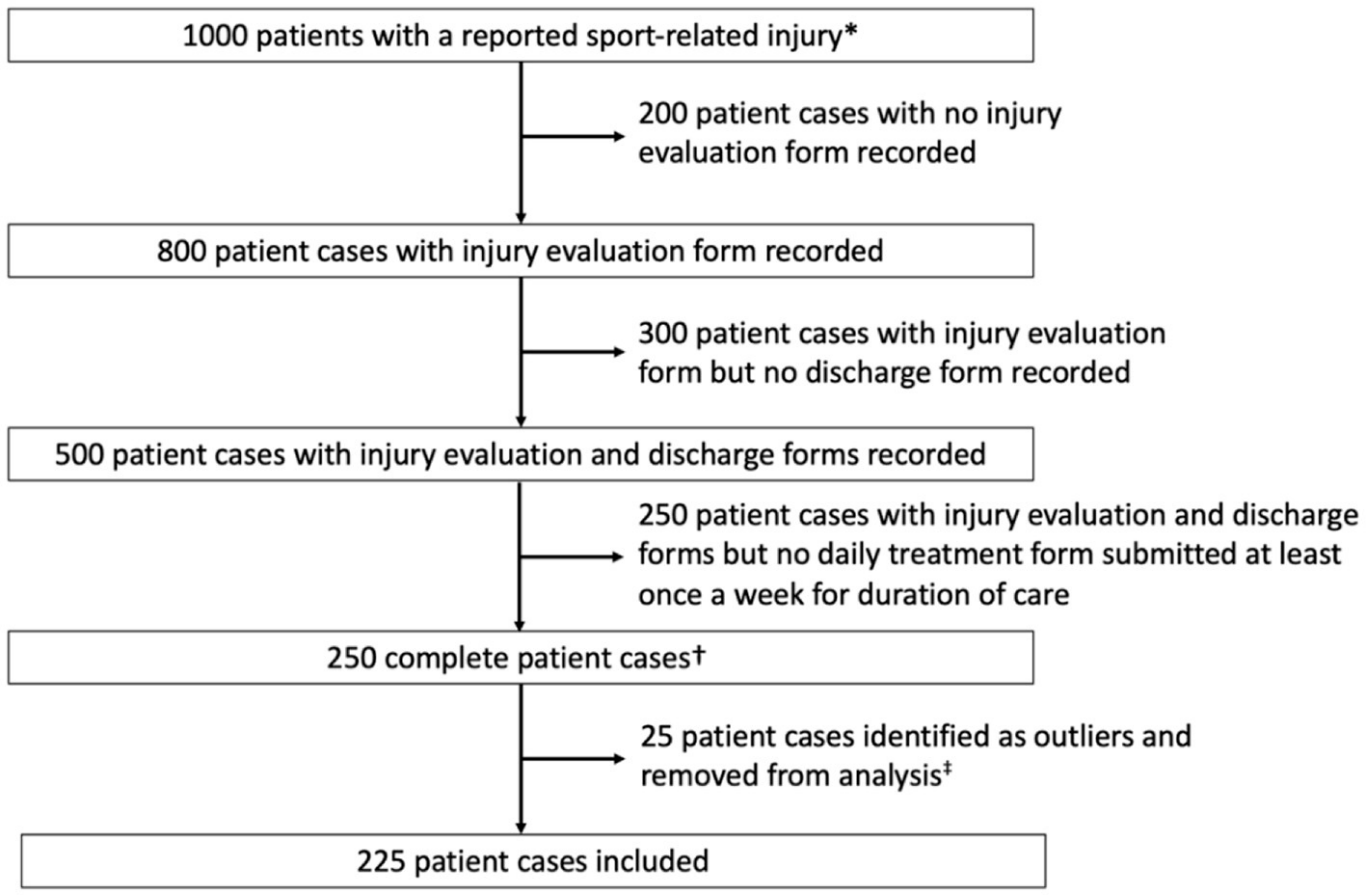
A flow diagram for selection of study cohort using mock data. *Identified by a submitted injury demographics form to the electronic medical record. ^†^Complete patient cases were operationalized as cases that had (1) an injury demographics form, (2) an injury evaluation, (3) daily treatment forms with at least one encounter per week for the duration of care, and (4) a discharge form. ^‡^Outliners were defined as patient cases that exceeded 1.5 times the interquartile range.

Relatedly, to provide the reader with a better understanding of the data, we have used the Strengthening the Reporting of Observational Studies in Epidemiology (STROBE) guidelines to enhance the transparency of our data reporting (Benchimol et al., 2015). For example, for the included patient cases, we have used a flow diagram to identify the number of patient cases at each stage of data processing and the number and reasons for exclusion of certain cases (Figure 2). This type of figure provides a clear and concise summary of how patient cases were identified and included for the study. Further, the step-by-step representation of the inclusion process provides the reader with a better understanding of the data entered into the EMR and the specific criterion by which cases were excluded from the final analysis (Benchimol et al., 2015). Together, this set of information provides better data transparency and more insight for readers to make an informed interpretation of the results.

In addition to missing time points, we also encountered data variability issues with the estimated cost study for knee injuries (Lam et al., 2021). While the criteria for complete patient cases helped to reduce data variability in the dataset, there were still outliers present in the dataset (Lam et al., 2021). For example, there were several patient cases that were treated for over 200 days while the median duration was reported to be closer to 25 days. Following previously described procedures (Mishra et al., 2019), we used the interquartile range for major variables (e.g., duration of care, number of visits) and identified cases that were 1.5 times the interquartile range as outliers. Outliers were excluded from the final analysis in an effort to provide a better estimate of typical clinical practice. Although in a more perfect situation the data would not be so varied, this is the nature of observational data. In publishing recent manuscripts, peer reviewers have been receptive to these approaches and consider them to be appropriate under the circumstances.

## Discussion and future directions

Refinement of methodological procedures is an inherent part of the scientific process. Other well-established groups such as the National Athletic Treatment, Injury and Outcomes Network (Dompier et al., 2015; Morris et al., 2020) and National Collegiate Athletic Association Injury Surveillance Program (Kerr et al., 2018) have reported similar needs to refine its processes surrounding data quality control and data management. These refinements have ranged from straightforward changes (e.g., the addition of new sports) to more complicated processes (e.g., dedicated quality control measures) (Kerr et al., 2018; Morris et al., 2020). As the AT-PBRN continues to grow and evolve, efforts to regularly improve the documentation (Bacon et al., 2017, 2018; Lam et al., 2020) and research processes remain a central focus of the research team.

While observational studies have provided clinicians and researchers with valuable and tangible information related to athletic health care, future efforts should look toward conducting experimental studies at the point-of-care (Lam et al., 2020). The point-of-care clinical trial is an approach that allows for clinical research to be feasibly conducted during routine clinical practice (Lam et al., 2020). As such, this design preserves the internal validity of traditional randomized controlled trials, minimizes bias found in observational designs, while taking advantage of the external validity and generalizability of a study conducted at the point-of-care.

Regardless of the specific research design, the value, relevance, and meaningfulness of clinical data will depend on the overall quality of data entered into the EMR. To advance the use of point-of-care data in athletic healthcare, a collective effort is needed in which established groups share ideas and best practices *via* methodological papers (Kerr et al., 2018; Morris et al., 2020) or perspectives such as this one. Through this type of crowdsourcing of information and lessons learned, progress can be made at a more rapid pace and better support the use of clinical data for patient care purposes.

## Data availability statement

The original contributions presented in the study are included in the article/supplementary material, further inquiries can be directed to the corresponding author/s.

## Author contributions

KL wrote the first draft of the manuscript. AM wrote sections of the manuscript. All authors contributed to conception and organization of this perspective and manuscript revision, read, and approved the submitted version.

## Conflict of interest

Author KL is the Director of the Athletic Training Practice-Based Research Network (AT-PBRN), which serves as the basis for this perspective. Also, authors KL, AS, TV, and AM hold a role of “scientist” within the AT-PBRN.

## Publisher's note

All claims expressed in this article are solely those of the authors and do not necessarily represent those of their affiliated organizations, or those of the publisher, the editors and the reviewers. Any product that may be evaluated in this article, or claim that may be made by its manufacturer, is not guaranteed or endorsed by the publisher.

## References

[B1] Bacon C. E. W. Eppelheimer B. L. Kasamatsu T. M. Lam K. C. Nottingham S. L. (2017). Athletic trainers' perceptions of and barriers to patient care documentation: a report from the Athletic Training Practice-Based Research Network. J Athl Train. 52, 667–675. 10.4085/1062-6050-52.3.15 28574752 PMC5517122

[B2] Bacon C. E. W. Kasamatsu T. M. Lam K. C. Nottingham S. L. (2018). Future strategies to enhance patient care documentation among athletic trainers: a report from the Athletic Training Practice-Based Research Network. J Athl Train. 53, 619–626. 10.4085/1062-6050-298-17 29893602 PMC6089026

[B3] Benchimol E. I. Smeeth L. Guttmann A.. (2015). The REporting of studies Conducted using Observational Routinely-collected health Data (RECORD) statement. PLoS Med. 12, e1001885. 10.1371/journal.pmed.1001885 26440803 PMC4595218

[B4] Bohr A. D. Aukerman D. F. Harmon K. G. Romano R. Hernández T. D. Konstantinides N.. (2021). Pac-12 CARE-Affiliated Program: structure, methods and initial results. BMJ Open Sport Exerc Med. 7, e001055. 10.1136/bmjsem-2021-001055 34079621 PMC8137172

[B5] Casey J. A. Schwartz B. S. Steward E. F. Adler N. E. (2016). Using electronic health records for population health research: a review of methods and applications. Annu Rev Public Health. 37, 61–81. 10.1146/annurev-publhealth-032315-021353 26667605 PMC6724703

[B6] Concato J. Lawler E. V. Lew R. A. Gaziano J. M. Aslan M. Huang G. D. (2010). Observational methods in comparative effectiveness research. Am J Med. 123:e16–23. 10.1016/j.amjmed.2010.10.004 21184862

[B7] Cowee K. Simon J. E. (2019). A history of previous severe injury and health-related quality of life among former collegiate athletes. J Athl Train. 54, 64–69. 10.4085/1062-6050-377-17 30657720 PMC6410981

[B8] Cowie M. R. Blomster J. I. Curtis L. H. Duclaux S. Ford I. Fritz F.. (2017). Electronic health records to facilitate clinical research. Clin Res Cardiol. 106, 1–9. 10.1007/s00392-016-1025-6 27557678 PMC5226988

[B9] Dompier T. P. Marshall S. W. Kerr Z. Y. Hayden R. (2015). The National Athletic Treatment, Injury and Outcomes Network (NATION): methods of the surveillance program, 2011-2012 through 2013-2014. J Athl Train. 50, 862–869. 10.4085/1062-6050-50.5.04 26067620 PMC4629944

[B10] Green L. White L. Barry H. Nease D. Jr Hudson B. (2005). Infrastructure requirements for practice-based research networks. Ann Fam Med. 3, S5–S11. 10.1370/afm.299 15928219 PMC1466956

[B11] Kerr Z. Y. Comstock R. D. Dompier T. P. Marshall S. W. (2018). The first decade of web-based sports injury surveillance (2004-2005 through 2013-2014): methods of the National Collegiate Athletic Association Injury Surveillance Program and High School Reporting Information Online. J Athl Train. 53, 729–737. 10.4085/1062-6050-143-17 30024769 PMC6188084

[B12] Lam K. Snyder Valier A. Bay R. (2016a). Changes in self-report of impairments, function and disability following sport-related knee injuries: a report From the Athletic Training Practice-Based Research Network. J Athl Train. 51, S142.

[B13] Lam K. C. Bacon C. E. W. Sauers E. L. Bay R. C. (2020). Point-of-care clinical trials in sports medicine resarch: identifying effective treatment interventions through comparative effectiveness resarch. J Athl Train. 55, 217–228. 10.4085/1062-6050-307-18 31618071 PMC7093921

[B14] Lam K. C. Marhsall A. N. Welch Bacon C. E. Valovich McLeod T. C. (2021). Cost and treatment characteristics for sport-related knee injuries managed by athletic trainers: a report from the Athletic Training Practice-Based Research Network. J Athl Train. 56, 922–929. 10.4085/1062-6050-0061.20 33237998 PMC8359723

[B15] Lam K. C. Marshall A. N. Holland B. Bay R. C. Wikstrom E. A. Snyder Valier A. R. (2022). Patients experience significant and meaningful changes in self-report of function during the first two weeks after an ankle sprain injury: a report from the Athletic Training Practice-Based Research Network. J Sport Rehabil. 1–7. 10.1123/jsr.2022-0014 35926848

[B16] Lam K. C. Snyder Valier A. R. Valovich McLeod T. C. (2015). Injury and treatment characteristics of sport-specific injuries sustained in interscholastic athletics: a report from the athletic training practice-based research network. Sports Health. Jan 7, 67–74. 10.1177/1941738114555842 25553215 PMC4272697

[B17] Lam K. C. Valier A. R. Anderson B. E. McLeod T. C. (2016b). Athletic training services during daily patient encounters: a report from the Athletic Training Practice-Based Research Network. J Athl Train. Jun 2 51, 435–41. 10.4085/1062-6050-51.8.03 27315222 PMC5076279

[B18] Marshall A. N. Kikugawa T. M. Lam K. C. (2019). Patient, treatment and cost characteristics associated with sport-related ankle sprains: a report from the Athletic Training Practice-Based Research Network. Athl Train Sports Health Care. 12, 173–180. 10.3928/19425864-20190521-0133237998

[B19] Marshall A. N. Lam K. C. (2020). Research at the point of care: using electronic medical record systems to generate clinically meaningful evidence. J Athl Train. 55, 205–212. 10.4085/1062-6050-113-19 31935140 PMC7017890

[B20] Mishra P Pandey CM Singh U Gupta A Sahu C Keshri A. (2019). Descriptive statistics and normality tests for statistical data. Ann. Card Anaesth. 22:67–72. 30648682 10.4103/aca.ACA_157_18 PMC6350423

[B21] Morris S. N. Chandran A. Wasserman E. B. Quetant S. L. Robison H. J. Collins C. (2020). Methods of the National Athletic Treatment, Injury and Outcomes Network Surveillance Program (NATION-SP), 2014-2015 through 2018-2019. J Athl Train. 56, 529–533. 10.4085/284-20 33150422 PMC8130775

[B22] National Athletic Trainers' Association. Athletic Training. https://www.nata.org/about/athletic-training (accessed June 22, 2022).

[B23] Rudin R. S. Friedberg M. W. Shekelle P. Shah N. Bates D. W. (2020). Getting value from electronic health records: research needed to improve practice. Ann Intern Med. 172:S130–S136. 10.7326/M19-0878 32479182

[B24] Sauers E. L. Valovich McLeod T. C. Bay R. C. (2012). Practice-based research networks, part I: clinical laboratories to generate and translate research findings into effective patient care. J Athl Train. 47, 549–556. 10.4085/1062-6050-47.5.11 23068593 PMC3465036

[B25] Seshadri D. R. Li R. T. Voos J. E. Rowbottom J. R. Alfes C. M. Zorman C. A.. (2019a). Wearable sensors for monitoring the physiological and biochemical profile of the athlete. NPJ Digit Med. 2, 72. 10.1038/s41746-019-0150-9 31341957 PMC6646404

[B26] Seshadri D. R. Li R. T. Voos J. E. Rowbottom J. R. Alfes C. M. Zorman C. A.. (2019b). Wearable sensors for monitoring the internal and external workload of the athlete. NPJ Digit Med. 2, 71. 10.1038/s41746-019-0149-2 31372506 PMC6662809

[B27] Valovich McLeod T. C. Kostishak N. Jr. Anderseon B. E. Welch Bacon C. E. Lam K. C. (2019). Patient, injury, assessment, and treatment characteristics and return-to-play timelines after sport-related concussion: an investigation from the Athletic Training Practice-Based Research Network. Clin J Sport Med. 29, 298–305. 31241532 10.1097/JSM.0000000000000530

[B28] Valovich McLeod T. C. Lam K. C. Bay R. C. Sauers E. L. Snyder Valier A. R. (2012). Athletic Training Practice-Based Research N. Practice-based research networks, part II: a descriptive analysis of the athletic training practice-based research network in the secondary school setting. J Athl Train. 47, 557–66. 10.4085/1062-6050-47.5.05 23068594 PMC3465037

